# Changes of the tRNA Modification Pattern during the Development of *Dictyostelium discoideum*

**DOI:** 10.3390/ncrna7020032

**Published:** 2021-05-28

**Authors:** Anne Hoffmann, Lieselotte Erber, Heike Betat, Peter F. Stadler, Mario Mörl, Jörg Fallmann

**Affiliations:** 1 Bioinformatics Group, Department of Computer Science, Interdisciplinary Center for Bioinformatics, Leipzig University, Härtelstraße 16-18, D-04107 Leipzig, Germany; anne.hoffmann@helmholtz-muenchen.de (A.H.); peter.stadler@bioinf.uni-leipzig.de (P.F.S.); 2 Helmholtz Institute for Metabolic, Obesity and Vascular Research (HI-MAG) of the Helmholtz Zentrum München at Leipzig University and University Hospital Leipzig, Philipp-Rosenthal-Str. 27, D-04103 Leipzig, Germany; 3 Institute for Biochemistry, Leipzig University, Brüderstraße 34, D-04103 Leipzig, Germany; lieselotte.erber@uni-leipzig.de (L.E.); heike.betat@uni-leipzig.de (H.B.); mario.moerl@uni-leipzig.de (M.M.); 4 German Centre for Integrative Biodiversity Research (iDiv) Halle-Jena-Leipzig, Competence Center for Scalable Data Services and Solutions, and Leipzig Research Center for Civilization Diseases, Leipzig University, D-04103 Leipzig, Germany; 5 Max Planck Institute for Mathematics in the Sciences, Inselstraße 22, D-04103 Leipzig, Germany; 6 Institute for Theoretical Chemistry, University of Vienna, Währingerstraße 17, A-1090 Wien, Austria; 7 Facultad de Ciencias, Universidad Nacional de Colombia, 111321 Bogotá, D.C., Colombia; 8 Santa Fe Institute, 1399 Hyde Park Rd., Santa Fe, NM 87501, USA

**Keywords:** *Dictyostelium discoideum*, transfer RNAs, fruiting body development, chemical modifications, tRNA sequencing, LOTTE-seq, immature reverse transcriptase arrest

## Abstract

*Dictyostelium discoideum* is a social amoeba, which on starvation develops from a single-cell state to a multicellular fruiting body. This developmental process is accompanied by massive changes in gene expression, which also affect non-coding RNAs. Here, we investigate how tRNAs as key regulators of the translation process are affected by this transition. To this end, we used LOTTE-seq to sequence the tRNA pool of *D. discoideum* at different developmental time points and analyzed both tRNA composition and tRNA modification patterns. We developed a workflow for the specific detection of modifications from reverse transcriptase signatures in chemically untreated RNA-seq data at single-nucleotide resolution. It avoids the comparison of treated and untreated RNA-seq data using reverse transcription arrest patterns at nucleotides in the neighborhood of a putative modification site as internal control. We find that nucleotide modification sites in *D. discoideum* tRNAs largely conform to the modification patterns observed throughout the eukaroytes. However, there are also previously undescribed modification sites. We observe substantial dynamic changes of both expression levels and modification patterns of certain tRNA types during fruiting body development. Beyond the specific application to *D. discoideum* our results demonstrate that the developmental variability of tRNA expression and modification can be traced efficiently with LOTTE-seq.

## 1. Introduction

Transfer RNAs (tRNAs) are specific adapter molecules that are essential for translating the mRNA-triplet code into the amino acid sequence of a nascent protein [1]. For this, tRNAs require a special conserved structure, characterized by a cloverleaf shape in the secondary and by an inverted “L” shape in the tertiary structure. The maturation process of tRNAs is complex and well-regulated including trimming, splicing of introns, editing, addition of a 3′-CCA end (if not encoded) and the modification of nucleotides [2]. With more than 90 different types of modifications identified, tRNAs represent a hot spot for this maturation step [3], with potential pathological effect known as “RNA modopathies”, see Suzuki [4] for a recent review. Modifications differ between nuclear und mitochondrial tRNAs, with modifying enzymes for mt-tRNAs typically being of bacterial origin [5]. Modifications of tRNAs act as checkpoints for tRNA integrity, regulate protein translation, modulate the structural stability and ensure the correct folding of the linear tRNA molecule into its three-dimensional shape. They are also involved in the structural fine-tuning of local elements and instigate a rapid adaptation of tRNAs to environmental changes, such as stress and temperature [6,7]. In addition, tRNA modification can contribute to regulation of tissue and cell development by influencing the translation efficiency [8,9].

Nucleotide modifications may affect reverse transcription (RT), a crucial step during the preparation of tRNAs for high-throughput sequencing, and thus become visible in RNA-seq data sets in different ways. While several modifications do not cause any changes in the cDNA sequencing pattern, others result in a position-specific increase of sequencing errors. It is also frequent that a modification blocks the complementary base-pairing interaction during cDNA synthesis, which is visible as an accumulation of immature RT arrest (RTa) at the position before the modified base [10,11]. The RT can also skip the modified nucleotide resulting in so-called *jumps* [12,13,14,15]. These events in cDNA synthesis represent *RT signatures* that vary by the type of modification, by the nature of the penultimate base encountered by the RT enzyme before interfering with the modified RNA residue [16], and by the RT enzyme [12], among other factors. 1-methyladenosine ($m^{1}A$) is the most prominent modification and is directly visible as conspicuous accumulation of mismatches and RTa sites in mapped RNA-seq data [16]. This particular modification of adenine is typically interpreted by Illumina sequencers as an A-to-T transversion or an A-to-G transition [13]. Furthermore, A-to-I (inosine) editing is also directly visible in RNA-seq data. While a non-edited adenine residue pairs with a thymine during reverse transcription, inosine pairs with cytosine, leading to an apparent A-to-G mismatch compared to the reference sequence [17]. Other modifications such as 1-methylguanosine ($m^{1}G$), $N^{2}$-methylguanosine ($m^{2}G$), or $N^{2},N^{2}$-dimethylguanosine (${m^{2}}_{2}G$) are also marked by increased mismatch rates. It was also reported that several methylations, e.g., $m^{1}G$, $m^{2}G$, ${m^{2}}_{2}G$, and 3-methylcytidine ($m^{3}C$) lead to premature RTa [18,19]. In some cases, modifications of the same type at different positions vary in the details of their RT signatures. In particular, $m^{1}G$ and dihydrouridine (D) may not always show significant levels of base misincorporation. Therefore, the combined observation of RTa sites and misincorporation is an effective tool for modification detection and classification.

Quantitative statements about true RTa signals are only possible for sufficiently high tRNA read coverage. Standard RNA-seq methods such as ribo-minus RNA-seq (rmRNA-seq) or total RNA-seq only capture a relatively small amount of tRNA sequences [20]. For example, rmRNA-seq yields only 0.9% short ncRNAs, which is usually insufficient to call modified nucleotides or quantifying expression. In contrast, LOTTE-seq [21] specifically hybridizes a DNA hairpin adapter to the tRNA 3′-CCA end. The ligation reaction catalyzed by T4 DNA ligase [22] accepts only complete CCA ends and efficiently excludes substrates with only a partial or no CCA end. Reverse transcription prior to the ligation of a second adapter allows including prematurely terminated cDNA products, increasing the number of tRNA reads and enabling the systematic detection of RTa sites. LOTTE-seq produces nearly exclusively (>98%) mappable tRNA reads [21]. The resulting high coverage of tRNAs makes it possible to reliably identify certain RT signatures.

A general problem with using second-generation RNA-seq data for modification detection is the need to distinguish chemical modifications from other causes of disagreement between read and reference sequence. Pyrimidine and A bonds are very sensitive to nuclease cleavage and the characteristics of specific RNA secondary structures can also cause RTa. An RTa site alone therefore cannot be always taken as conclusive proof of a modified residue [12]. A recent tRNA sequencing method, mim-tRNAseq [23], is optimized for read-through in full-length tRNA reads and thus can focus entirely on misincorporation patterns. Mismatches also deriving from other sources, however, further complicate the identification of modifications, e.g., PCR errors accumulate during amplification and incorrect bases are called during the sequencing step. After read mapping to the reference sequence, these errors are visible as mismatches or indels. Additional mismatches and indels may occur during read mapping due to alignment errors and the genome sequence itself. Since these errors cannot be fully controlled during library preparation, sequencing, and read mapping, noise must be separated from true signals during the modification calling step [24]. The situation for tRNAs is further complicated by the presence of very similar but not absolutely identical paralogous genes that require specialized read mapping strategies to distinguish variations between variants from signatures of chemical modifications [23,25].

In current protocols this is achieved by comparing chemically treated data with untreated controls [18,26,27,28]. Chemical treatments are unavoidable for the detection of modifications such as 7-methylguanosine ($m^{7}G$) [28,29] and 5-methylcytidine ($m^{5}C$) [30,31,32] that do not affect RT, and to provide conclusive evidence for the chemical nature of the modification. Nevertheless, it is possible to detect a large class of modifications directly from untreated sequencing data. Instead of an unmodified sample, the sequence neighborhood of a putative modification site in the same molecule is used as an internal control. Wang et al. [33] present a dedicated experimental and analysis procedure for *Escherichia coli* based on sample pooling and application of restrictive cutoffs. The distribution of nucleotide mismatch and RTa, respectively, can be used not only to identify modified sites but also to predict the identity of the modification; such a mismatch-based method is implemented in HAMR [11,15]. While this approach cannot provide unambiguous proof for the identity of a modification, it can be used as evidence in particular in comparative settings, e.g., when the identity of the modification at a homologous site in another species has been validated e.g., by mass spectrometry [34,35]. While approximately complete profiles of tRNA modifications are not available for most organisms, they are known for a limited number of eukaryotes including yeast, human, and rat [3]. Despite the intrinsically lower level of confidence, the direct detection approach also has distinctive advantages. In particular, it is much less costly and it can be applied opportunistically to RNA-seq data that have been produced for other purposes, such as the quantification of tRNA pool compositions [19,21,36]. Direct detection is particularly well-suited for tracking quantitative changes of modification patterns across time courses or tissues, because it avoids the introduction of additional technical variation due to the chemical treatment of independent samples.

**Figure 1:**
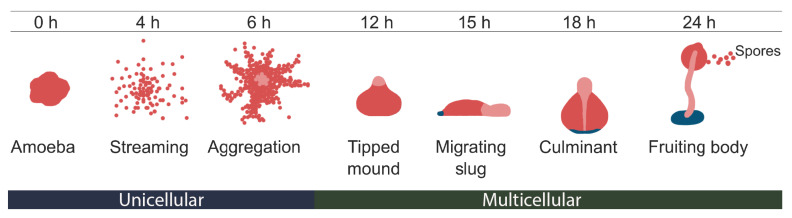
Life cycle of *Dictyostelium discoideum*. Developmental morphogenesis of *D. discoideum* starting from a single and vegetative amoebae (0 h). Aggregation of the single amoebae is mediated by the chemotaxis of cells to form a multicellular aggregate (6 h after starvation). During this process, multicellular aggregates stream toward a central domain or aggregation center. Aggregation results in the formation of a mound (multicellular organism, 12 h after starvation). Mound forms a tipped mound (14 h after starvation). The tip extends and forms a finger which might fall over to form a phototactic migrating slug (16 h after starvation) or begins culmination (20 h after starvation) to form a fruiting body. Finally, the fruiting body contains a sorus of spores on top of a stalk which germinate following dispersal, renewing the cycle (24 h after starvation). The figure ist modified from [46].

Here, we investigate the developmental time course of tRNA expression and modification patterns of *Dictyostelium discoideum* using LOTTE-seq. *D. discoideum* is a single celled amoeba and widely used model organism that develops a multicellular organism with differentiated cell types under starving conditions [37], see Figure 1. When the main food source, bacteria, becomes scarce, *D. discoideum* cells release cAMP, triggering cell aggregation and development [38,39]. The arising fruiting body contains spores that are released under optimal growth conditions [39]. This complex development from a unicellular to a multicellular organism is accompanied by extensive alterations of the gene expression pattern, such as the up-regulation of development-specific genes and down-regulation of mitochondrial and metabolic genes [40,41]. Non-coding RNAs are also involved in regulation of the development [42]. Not much is known about the alteration of the composition or the modification pattern of the tRNA pool during the development of *D. discoideum*, even though the tRNAs of *D. discoideum* were initially investigated by Dingermann et al. in the 1970s [43,44,45]. We demonstrate here (1) that RTa and misincorporation sites of *D. discoideum* obtained by LOTTE-seq are consistent with the typical modified nucleotides found in other eukaryotes and (2) that modifications exhibit specific dynamics during development.

## 2. Results

### 2.1. tRNA Modification Diversity in D. discoideum

*D. discoideum* contains over 400 tRNA genes with an unusual and unique distribution pattern throughout the genome, partially as pairs or triplets with identical anticodons [47]. To investigate tRNA modification patterns in *D. discoideum* in a systematic manner, individual tRNA pools were analyzed for candidate modification sites by their RT signatures (accumulation of base misincorporations and/or apparent RTa sites) extracted from LOTTE-seq data. To this end, we elaborated on the knowledge about modification-specific RT signals provided by a series of previous studies [3,11,12,13,16,18,25,26,28,34,48], adding our own observations as well as published information on the TGIRT enzyme [19,49,50]. This group II intron-derived thermostable reverse transcriptase exhibits a high fidelity and processivity [51]. Yet, it produces RT signals at certain nucleotide modifications [19,49,50]. This allows for an easier classification of tRNA modification patterns from analysis of RT-based RNA-seq data, especially methylations [49]. Table S1 summarizes our improved collection of common tRNA modifications visible in untreated RNA-seq data and describes how these modifications are detectable. Since some RT signatures fit to several modification types, an unambiguous assignment without prior knowledge is challenging. Therefore, we incorporated information compiled in the tRNAmodviz database [3] to classify specific modification patterns by incorporating the knowledge about chemical modifications of homologous tRNA positions in other species. Yet, analysis of the RT signatures alone cannot exclude the possibility of misclassifications or false positive hits resulting from, e.g., robust secondary structures. To be able to classify the modifications unambiguously, specific chemical treated RNA-seq data or mass spectrometry analyses would be necessary [35,52,53]. Here, we are content with well-founded hypotheses concerning the identity of the modifications and focus on the variations of modification patterns across the developmental time course.

Since tRNAs are subject to concerted evolution, many of the genes code for identical or nearly identical mature tRNAs. We therefore clustered the 421 individual tRNAs of *D. discoideum* into 70 (55 cytosolic tRNA and 15 mitochondrial tRNA (mt-tRNA)) distinct groups of identical sequences as described in [25]. Since reads are mapped to tRNA clusters, all analysis pertains to these groups of in practice indistinguishable mature tRNAs rather than to individual tRNAs. Only about 5% of the tRNA reads correspond to mt-tRNAs. Their detected modifications are listed in Table S3. Due to their comparably low abundance we do not discuss them further.

**Figure 2:**
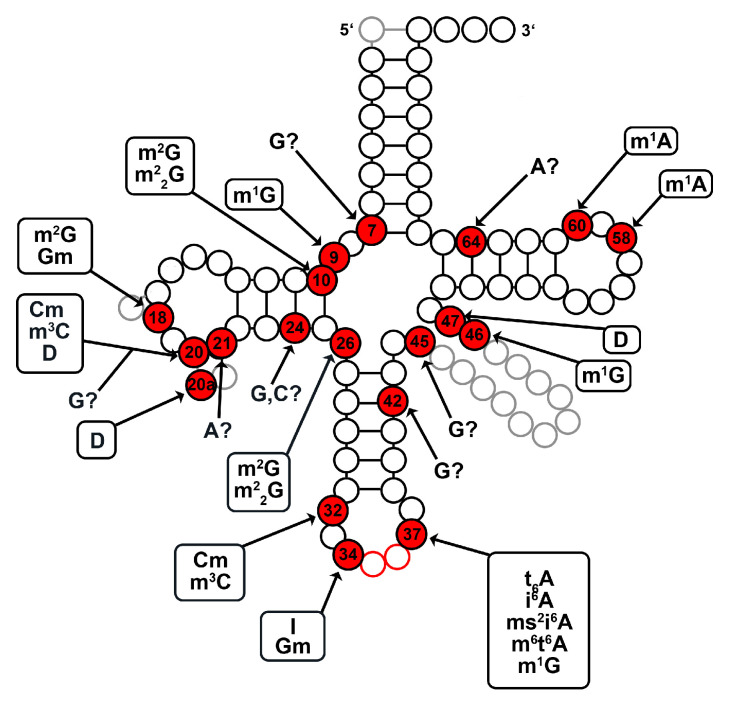
Modification pattern of *Dictyostelium discoideum* tRNAs. All tRNA positions showing accumulations of base misincorporations and/or RTa sites are highlighted in red. Modifications that matched to known tRNA modifications of other species from the tRNAmodviz database [3] are highlighted at the individual tRNA positions. Modifications, for which no clear classification is possible based on unambiguous RT signatures and no information is available from other species, are marked with a question mark next to the affected nucleotide. The tRNA positions are numbered according to Sprinzl et al. [54]). Abbreviations: A–adenosine; C–cytidine; Cm–$2^{\prime }-O$-methylcytidine; D–dihydrouridine; G–guanosine; Gm–$2^{\prime }-O$-methylguanosine; I–inosine; $i^{6}A$–$N^{6}$-isopentenyladenosine; $t^{6}A$–$N^{6}$-threonylcarbamoyladenosine; U–uridine; ${\mathrm{ms}}^{2}i^{6}A$–2-methylthio-$N^{6}$-isopentenyladenosine; $m^{1}A$–1-methyladenosine; $m^{1}G$–1-methylguanosine; $m^{1}I$–1-methylinosine; $m^{2}G$–$N^{2}$-methylguanosine; ${m^{2}}_{2}G$–$N^{2},N^{2}$-dimethylguanosine; $m^{3}C$–3-methylcytidine; $m^{6}t^{6}A$–$N^{6}$-methyl-$N^{6}$-threonylcarbamoyladenosine.

The observed signals for modification summarized over all time points are shown in Figure 2, detailed data can be found in Table S2. In general, we observed 19 modified tRNA positions (7, 9, 10, 18, 20, 20a, 21, 24, 26, 32, 34, 37, 42, 45, 46, 47, 58, 60, and 64; numbering according to Sprinzl et al. [54]). Our analysis allows the unambiguous classification of most of the modified tRNA residues to methylations such as $m^{1}G$ (G9, G37, G46) and $m^{1}A$ (A58, A60). Furthermore, we also observed several other modifications as for example inosine, which is supposedly undetectable with our RTa approach, at position 34 and dihydrouridine at positions 20, 20a and 47. In Findeiß et al. [13], it was demonstrated that $2^{\prime }$-*O*-methylcytidine (Cm) and $2^{\prime }$-*O*-methylguanosine (Gm) are indeed recognizable in RNA-seq data on the basis of RTa and misincooperation sites, albeit with considerably weaker RT signals due to the standard RNA-seq approach. Through the increased sensitivity acquired via LOTTE-seq we should be able to detect RT signals for Cm (e.g., C32) and Gm (e.g., G34) modifications. However, this further complicates the unambiguous classification of modifications based solely on their RT signatures and intensities. For instance, a modified cytidine at tRNA positions 20 and 32 may be either $m^{3}C$ or Cm. The same applies to modified guanosine residues, which could be classified to $m^{2}G$ and Gm at position 37. Unambiguous classification is also not possible for other tRNA modification, especially for modifications that display nearly identical RT signatures known for these residues in other species. For example, G10 and G26 modifications could probably be $m^{2}G$ or ${m^{2}}_{2}G$ modifications. It is also indistinguishable whether modified adenosines (A37) refer to $N^{6}$-threonylcarbamoyladenosine ($t^{6}A$), $N^{6}$-isopentenyladenosine ($i^{6}A$), 2-methylthio-$N^{6}$-isopentenyladenosine (${\mathrm{ms}}^{2}i^{6}A$), or $N^{6}$-methyl-$N^{6}$-threonylcarbamoyladenosine ($m^{6}t^{6}A$). In addition, we profiled modifications at positions G7, G20, A21, C24, G24, G42, G45, and A64, where no modifications are known in other species of the tRNAmodviz database. Depending on the RT signature, different types of tRNA modifications may be applicable, as listed in Table S1.

We also processed our data with HAMR (Table S3), which accounts for nucleotide misincorporation but does not evaluate RTa site enrichment. Considering the time point 0h after starvation as example, the mismatch-based HAMR methods recovers 22% of the modified tRNA positions detected by our method applying a stringent parameter setting. With a less stringent and more sensitive set, HAMR detects up to 31%. In addition, HAMR recognized the following modifications with the stringent parameter set at time 0 h: D20 (18 times), $m^{3}C$31 (6 times), and D47 (1 times), and additional D20 (29 times) modifications with the more sensitive parameter set, both parameter settings are explained in Section 4.3. The classifications of RT signatures by HAMR are consistent with most of our findings, although we encountered also some discrepancies with the HAMR results. For example, for tRNA position T20 and T47 only D modifications have been described in the literature [3]. While HAMR recognizes this correctly for most tRNA types (e.g., His${}_{20}$GTG) it classifies the modification as pseudouridine (Ψ) e.g., in Glu${}_{20}$TTC and Ala${}_{47}$AGC. This also occurs sometimes within the same tRNA at different developmental stages. For instance, Gly${}_{20}$GCC is assigned to D at 0 h, while it is classified to Ψ at 6 h. Furthermore, Pro${}_{37}$TGG and Leu${}_{37}$TAA show very similar signatures of comparable intensity (G-to-T mismatches and strong accumulation of RTa sites). HAMR classified the Pro${}_{37}$TGG modification as $m^{1}G$, while the signal at Leu${}_{37}$TAA is classified as $m^{2}G$|${m^{2}}_{2}G$. We suspect that this is explained by the inclusion of base pairing properties in the classifier of HAMR, given that tRNAmodviz only reports $m^{1}G$ for this site.

Our results indicate a complex pattern of modifications for the tRNAs in *D. discoideum* comparable to those found in other Eukaryotes. We can, at this timepoint, only speculate about the specific functions of the newly discovered tRNA modifications in *D. discoideum*. They might contribute to correct folding of the tertiary structure or might be involved in the recognition by aminoacyl tRNA synthetases [55,56].

### 2.2. tRNA Modifications Vary during the D. discoideum Life Cycle

**Figure 3:**
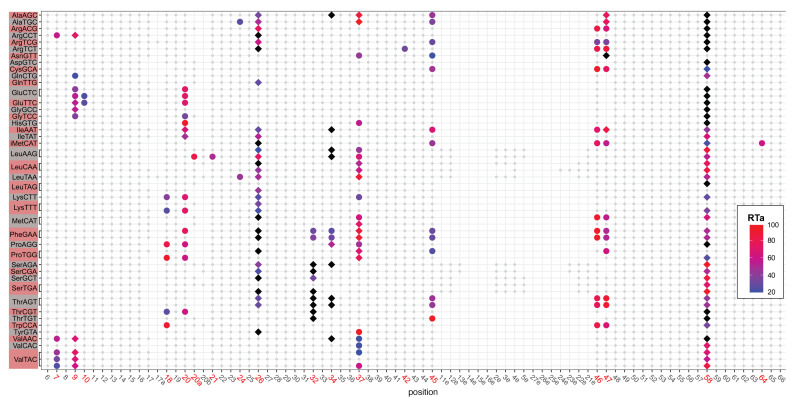
Overview of modification patters in *Dictyostelium discoideum* tRNAs shown for 0h after starvation as an example. The tRNAs are ordered by isoacceptor family and anticodon with one line for each distinct tRNA sequence and aligned according to Sprinzl’s nomenclature for nucleotide positions [54]. Gray background dots indicate nucleotides present in a sequence. Colored marks indicate the RTa intensities (fraction of reads that stop at position n + 1). Colored ⧫ symbolizes tRNA positions that show both RTa and misincoorperation sites, while the symbol in black refers to tRNA positions that show only misincoorperations. • show positions derived by their RTa profile. Only tRNAs with detectable modifications are included. The full set and other time points are listed in Table S2.

A recent study on *Oryza sativa* and *Arabidopsis thaliana* demonstrates that the modification level of several methylated tRNA genes differ significantly between different stages of development [57]. In *D. discoideum*, Schachner et al. provided evidence that tRNAs were modified in a development-specific manner already in 1984 [58]. Furthermore, tRNA modifications are known to be involved in the development of certain tissues in *A. thaliana* leafs [8] and in cell differentiation [9]. Thus, we investigated changes of tRNA modification patterns during the *D. discoideum* development. We used LOTTE-seq data for the different time points 0, 6, 16, 20 and 24 h after starvation (Figure 1). Modification profiles for tRNA cluster at 0 h is given in Figure 3 and the remaining time points are available in Figures S1–S4.

**Figure 4:**
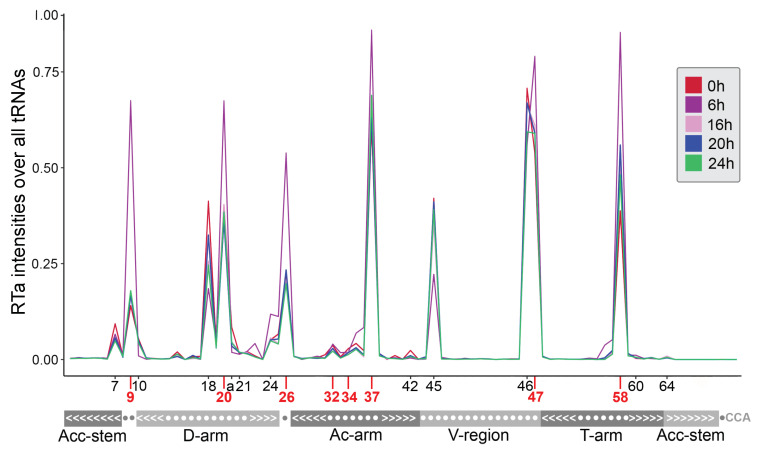
RTa distribution during the life cycle of *Dictyostelium discoideum* (0, 6, 16, 20, and 24 h). The number of RTa intensities is summarized over all tRNAs and normalized over all reads at each standard tRNA position *n* (x-axis) (numbered according to Sprinzl et al. [54]). Only RTa sites with an intensity ≥ 20% at the specified position in at least one of the tRNA clusters were considered and are highlighted in black. tRNA positions that show (additional) base-calling mismatches are highlighted in red on the x-axis. The secondary structure of tRNAs is given in dot-bracket notation (bottom).

In each developmental stage, the same eight tRNA positions (9, 20, 26, 32, 34, 37, 47, and 58) show accumulations of base misincorporations regarding the general modification profile. However, a dynamic pattern over the investigated time points of development becomes apparent when RTa intensities are considered (Figure 4). The highest accumulation of RTa compared to the remaining analyzed developmental stages can be observed at 6h after starvation, when the multicellular organism starts to form. Here, positions 9, 20, 24, 26, 37, 47, and 58 show the highest RTa intensities (fraction of reads that stop at position n+1) measured over all tRNAs, indicating a high abundance of potentially specific modifications at this developmental stage. For positions 10, 18, 20a, and 45 the intensities of RTa is lowest at 6h compared to the RTa of the other investigated time points. Interestingly, the RTa intensity is approximately the same for the remaining developmental stages at some of these positions (9, 10, 20, 20a, 24, 26, 37, 45, and 47). However, dynamics in RTa profiles is also visible in other developmental stages at positions 18, 46, and 58. For example, the RTa intensities at 0h is strongest at position 18 and in turn lowest at position 58 compared to the other developmental stages, whereas the tRNAs at 24 h show the lowest relative amount of RTa at position 46. Conversely, the residual modified positions (7, 21, 32, 34, 42, 60, and 64) show no substantial differences in RTa measured over all tRNAs between the time points.

Considering individual tRNA genes and no longer the general modification profile, the investigated time points show differences in the number of tRNAs with modifications detected at certain positions (Table S2). At 6 h after starvation, the number of modified tRNAs differs most from the other investigated developmental stages, since they are largely reduced at certain positions (C20, T20, G26, and G45) or are absent (RTa intensity < 20%) at G7, G10, G20, T20a, A21, and G42. In general, at 0 h and 16 h after starvation, a significant increase in the occurrence of modified tRNAs is visible. In particular, a modified G at position 34 is only present at 0h and can be associated with 33 distinct tRNAs types. However, a similar number of altered tRNAs occurs at positions C24, A34, T47, and A58.

It is important to note here that the intensity of RTa does not correlate with the expression of modified residues, indicating differences between specific modifications that do not simply follow a global trend. For example, the number of individual tRNAs with modifications at T20 and G26 is significantly lower at 6 h compared to the other time points, although it displays the highest proportion of RTa measured over all tRNAs at these positions. To investigate whether the proportion of RTa correlates with codon usage, which would indicate an expression bias, we compared both for known positions of modifications. Figure S5 shows no indication for such a bias. Considering all time points and the mean relative fraction of reads per codon cluster per position which shows a modification in more than one codon cluster, we observe that the number of RTa sites is not significantly correlated with the expression of tRNAs. Thus, RTa levels are not confouded by expression levels. Together with the fact that RTa sites are not equally distributed across the tRNA sequence we conclude that a pileup of RTa events (i.e., intensity) can be considered a valid indicator of modification.

Our results reveal drastic differences in the modification frequency at certain positions during development. In particular, the analysis of tRNA modifications 6 h after starvation (before reaching multicellularity) is interesting, as we observe an increase of methylations at several tRNA positions. This is in agreement with an increased level of mRNA, e.g., for the tRNA methyltransferase trmt61 (data retrieved from dictyExpress [59]) which increases 50-fold during development and is probably involved in the methylation of A58 [60]. Furthermore, up-regulation of the Dnmt2-homologue DnmA [61] and tRNA adenine-$N^{1}$-methyltransferase [62] was observed during the development of *D. discoideum*. Since the same tRNA species are not always modified at all investigated stages of the life cycle of *D. discoideum* and they differ in the number of RTa, we conclude that the modification profile of distinct tRNAs is regulated according to the needs at the particular stage of development, implying specific regulatory function of tRNA modifications during development is conceivable. A role of tRNA modification in the development was shown e.g., in *A. thaliana*, where tRNA modifications in and around the anticodon loop influence the development of leaf tissue [8].

We also observe an altered modification of tRNA positions in and around the anticodon loop, which could influence the translation during development in *D. discoideum*. For instance, the number of modified adenines is considerably lower at 20 h and 24 h compared to the other developmental stages, or G34 modifications which can only be found at 0 h after starvation. Another interesting speculation is the adoption of the tRNA modification in response to stress situation as was shown e.g., for *Oryza sativa* [57], *Saccharomyces cerevisiae* [63] and other organisms [64]. Thus, starvation might lead to an altered modification pattern in *D. discoideum* and contribute to translation regulation as postulated in [65]. Furthermore, starvation stress also leads to a depression of nutritional components. This was observed e.g., for queuine (Q), an important modified nucleotide which is mainly retrieved from their bacterial food source [58]. Under starvation, queuine levels decrease, leading to more extensive incorporation of guanine instead of queuosine [66], thus implicating an essential role of micronutrients for tRNA modification.

In summary we observed dynamic changes of tRNA modification levels, quantified by RTa levels. Overall, modification levels peak at 6 h while a complementary set show the lowest modification levels at the same time point. Changes in modification levels appear primarily in a site-specific rather than a tRNA-specific manner. This suggests that temporal differences in tRNA modifications are primarily driven by differences in abundance and/or activity of the modifying enzymes.

## 3. Discussion

We showed that many of the well-known chemical modifications can be detected in untreated RNA-Seq samples by their clear RT signatures, best integrating both RTa events and nucleotides misincorporations. Furthermore, the only requirement for such an analysis, high enough coverage of tRNA species, can be met easily with tailored NGS techniques including the LOTTE-Seq method used here. The combination of base misincorporation, RTa, and knowledge of modifications reported for homologous sites in other species makes it possible to detect and quantify modified bases and to produce well-founded hypotheses about their chemical nature. Of course, sequencing data alone cannot provide conclusive evidence about the type of modification. This would require dedicated validation experiments using MS methods [35] and/or the sequencing of RNA libraries after specific chemical treatments [26]. Nevertheless, sequencing data can provide useful insights on temporal or developmental changes in modification patterns. Here, we have shown that tRNA modifications in *D. discoideum* are mostly confined to sites that have been described in other species. RTa and misincorporation patterns are consistent with the types of modifications previously reported for these sites. Furthermore, we observed that the modification patterns exhibit systematic changes during *D. discoideum* development. To our knowledge, this is the first systematic investigation of tRNA modifications in *D. discoideum*.

Here we have used an analysis pipeline that considers both misincorporations and RTa information, see Section 4.3 for details. Since HAMR, the most widely used alternative tool, relies exclusively on misincorporation sites, it is dependent upon sequencing data that provide a sufficiently large number of read-through events. HAMR thus performs particularly well with data from full length tRNA sequencing or specialized methods such as mim-tRNA-seq. Conventional RNA-seq data and LOTTE-Seq, on the other hand, are enriched in RTa events and thus call for the use of methods that make use of RTa information. LOTTE-Seq enriches mature tRNA sequences irrespective of whether they are complete or incomplete, making it more difficult for HAMR to accumulate a sufficient amount of read-through data in particular towards the 5′-end of the tRNAs. In addition, we have shown here that the RTa sites provide useful additional information that increases the sensitivity and at the same time allows for a quantification of modification levels by measuring the relative intensity of RTa at a given candidate site.

The approach presented here enables economic high-throughput screens of tRNA-modification and therefore can provide a quick overview of the situation in poorly studied organisms. In particular, it can be decided quickly whether modification patterns are consistent with knowledge from related species, or whether there are unusual modified or unmodified positions that may warrant an in-depth characterization. A major advantage is that our methodology can also be applied to fortuitously available datasets that were generated with other biological questions in mind. Recent work in human cells suggested that differences in tRNA expression are not primarily reflected in the abundance of mature tRNAs, but rather lead to changes in the abundance of immature tRNAs and tRNA fragments [67]. A combination of LOTTE-seq and small-RNA-seq could quickly clarify whether this is true in general. Finally, it remains an interesting question for future research whether the enzymes responsible for chemical modifications in tRNAs are actively involved in regulating the composition of the pool of mature tRNAs.

## 4. Materials and Methods

### 4.1. Isolation of *D. discoideum* Total RNA

Total RNA was isolated from *D. discoideum* Ax2 cells of different developmental stages (0, 6, 16, 20 and 24 h after starvation) with TRIzol^®^ (Thermo Scientific) according to the manufacturer’s instructions. High-throughput sequencing of tRNAs with 3′-CCA end total RNA preparations from 0, 6, 16, 20 and 24 h after starvation were prepared for sequencing according to the tRNA-specific LOTTE-seq approach [21] with the exception that TGIRT (thermostable group II intron reverse transcriptase) was used for reverse transcription due to an increased yield of full-length cDNA.

### 4.2. tRNA Read Mapping

Demultiplexing of the sequenced reads were performed using bcf2fastq (v.2.2, https://support.illumina.com/downloads/bcl2fastq-conversion-software-v2-20.html, accessed on 9 March 2019), allowing 0 mismatches in the barcode sequences. Adapters of raw reads were trimmed applying Cutadapt (v1.16) [68]. Only reads which surpass a quality cutoff of 25, a maximum allowed error rate of 0.15 and a read length from 8 to 95 nts were retained. Standard pre- and post-trimming quality control was computed using FASTQC (v0.11.4) [69]. Annotation of pre- and mature tRNAs as well as the mapping of the high-throughput data were prepared on the basis of the best practice workflow for accurate mapping of tRNA reads, as previously described in [25]. In brief, tRNAs were annotated with tRNAscan-SE [70] and masked in the reference genome. tRNA sequences were extended by genomic sequences to estimated precursor tRNAs (pre-tRNAs) and added as additional contigs to the masked genome. To be able to detect modifications based on misincoorporations, reads were mapped to this modified “artificial” genome using segemehl v0.2.0-418 [71], requiring a minimum accuracy of 80% (with up to 20 mismatches allowed for a 100 nt long read). Anticipating a high density of modification-induced mismatches and short reads due to the nature of tRNA or RTa sites, we allowed a maximum of 3 mismatches in the segemehl seed regions, increased the e-value cut-off to 500 for seed extension, and considered at most 1000 mappings per seed. We note that the ESA-based read mapper segemehl defines seeds as maximal infixes of a read with a given bound on the discrepancies to the reference, which is then extended to a mapping of the entire read [72]. It can therefore also handle reads without long substrings that match exactly to the reference.

Mapped reads displaying specific hallmarks of pre-tRNAs such as the presence of 5′ leader sequences and all reads that did not map to the tRNA contigs were removed from further analysis. This initial mapping serves to reduce mapping artifacts caused by genome-transcriptome differences. In a second step, the remaining reads were mapped against a nonredundant set of mature tRNA sequences, which was obtained from the genomic tRNA sequences by removing introns, unifying identical sequences, and appending 3′-CCA tails. This avoids the handling of multiply mapped reads corresponding to distinct tRNA genes with identical sequences. Thus, only the uniquely mapped reads from this second step are retained for subsequent analysis. The genome of *D. discoideum* (dicty 2.7), downloaded from NCBI (Database resources of the National Center for Biotechnology Information 2016), served as reference.

### 4.3. Profiling of Modifications Sites in tRNAs

We consider chemical modification that cause RTa and/or mismatches in the mapping profile of untreated RNA-seq data. RTa is detectable as a sharp increase of read coverage in the sequence position following the modified nucleotide. In addition, misincorporations are frequently observed at the modified position itself [13,15]. To detect putative modifications, their misincorporation signal must be distinguished from random sequencing errors and mapping artifacts from true misincorporation sites. We used mpileup and call from bcftools (v1.9) [73] to identify sites with significant misincorporations. Variants below the Phred-scaled confidence threshold of 20 and hits with less than 10 reads were filtered out and excluded from further analysis. In addition, we profiled tRNA modifications based on apparent accumulations of RTa. As the TGIRT enzyme can occasionally cause weak accumulations of RTa in combination with a specific mismatch pattern up to three positions before the actual modification, which occurs frequently e.g., for $m^{3}C$ modifications, reads that produce such RTa signals and mismatches were manually filtered out to reduce false-positive signals. The *RTa intensity* at position *n* is defined as the total number of reads at position *n* divided by the total number of reads covering position n+1, therefore returning the fraction of reads stopping at position n+1, indication a modification at position *n*. As LOTTE-seq reads are derived from the 3′-end of a tRNA, an RTa event will decrease coverage towards the 5′-end of a tRNA. Only hits with a intensity of RTa greater or equal 20% compared to the amount of available reads at that position and a coverage of at least 10 reads were considered to be a modification signal. The detected modification sites were classified according to our collection of modification-specific TGIRT enzyme signals for the relevant modifications in *D. discoideum*, see Table S1. In addition, we compared the putative modifications to the modification information for all species provided by the tRNAmodviz database [3], since specific tRNA modifications for *D. discoideum* are not available.

We also applied HAMR [15] as an alternative approach for the identification of tRNA modifications with the following recommended parameter settings: minimum base calling quality score: 25; minimum read coverage of a genomic position: 10; expected percentage of mismatches based solely on sequencing error: 0.05; hypothesis to be tested: H4; maximum *p*-value cutoff: 0.01; maximum FDR cutoff: 0.05; minimum fraction of reads that do not differ from the reference sequence at a potentially modified position: 0.05. Since this parameter setting is optimized based on RNA-seq data from human and *Saccharomyces cerevisiae*, we additionally applied a less stringent setting to increase the sensitivity: maximum *p*-value cutoff: 1; maximum FDR cutoff: 1; minimum percentage of reads that must match the reference nucleotide: 0.01.

### 4.4. Fitting tRNAs to the Standard Model

All tRNA sequences were aligned against the tRNAdb database [74] to obtain secondary structure information applying blastn (v2.4.0) [75]. The highest scoring hits were selected to adjust for standard numbering of nucleotides in the tRNA sequences according to Sprinzl et al. [54].

## Data Availability

All data in this study are included in this published article and its supplementary information files. For each time course of *D. discoideum* raw RNA-seq data are available at https://www.ncbi.nlm.nih.gov/bioproject/PRJNA665588.

## Supplementary Materials

The following are available online at https://www.mdpi.com/article/10.3390/ncrna7020032/s1, Figure S1: Overview of modification patters at 6 h, Figure S2. Overview of modification patters at 16 h, Figure S3. Overview of modification patters at 20 h, Figure S4. Overview of modification patters at 24 h, Figure S5. Correlation plots between codon usage and RTa pileup, Table S1: Collection of reverse transcriptase signatures of tRNA modifications, Table S2. Differences regarding the number of modified tRNAs.

## Abbreviations

   The following abbreviations are used in this manuscript:

cAMP	cyclic adenosine monophosphate
Cm	2′-O-methylcytidine
D	dihydrouridine
ESA	enhanced suffix array
Gm	2′-O-methylguanosine
HAMR	high-throughput annotation of modified ribonucleotides
I	inosine
$i^{6}A$	$N^{6}$-isopentenyladenosine
LOTTE-seq	long hairpin oligonucleotide based tRNA high-throughput sequencing
miRNA	microRNA
mt-tRNA	mitochondrial tRNA
${\mathrm{ms}}^{2}i^{6}A$	2-methylthio-$N^{6}$-isopentenyladenosine
$m^{1}A$	1-methyladenosine
$m^{1}G$	1-methylguanosine
$m^{2}G$	$N^{2}$-methylguanosine
${m^{2}}_{2}G$	$N^{2},N^{2}$-dimethylguanosine
$m^{3}C$	3-methylcytidine
$m^{5}C$	5-methylcytidine
$m^{7}G$	7-methylguanosine
$m^{6}t^{6}A$	$N^{6}$-methyl-$N^{6}$-threonylcarbamoyladenosine
NCBI	Database resources of the National Center for Biotechnology Information
PCA	polymerase chain reaction
pre-tRNAs	precursor tRNAs
Q	queuine
rmRNA	ribo-minus RNA-seq
RNA-seq	RNA sequencing
RT	reverse transcription
RTa	reverse transcription arrest
$t^{6}A$	$N^{6}$-threonylcarbamoyladenosine
snoRNA	small nucleolar RNA
tRNA	transfer RNA
Ψ	pseudouridine
