# Supplementary material for: Changes of the tRNA Modification Pattern during the Development of Dictyostelium discoideum
Source: Noncoding RNA. 2021 May 28;7(2):32. doi: 10.3390/ncrna7020032 (PMC8163159; doi:10.3390/ncrna7020032)
Supplement: Supplementary file 1 [file ncrna-07-00032-s001.zip › Suppl/Supplement.pdf]

Article

Supplementary files: Changes of the tRNA modification pattern during the development of *Dictyostelium discoideum*

Anne Hoffmann[1,2,†], Lieselotte Erber[3,†], Heike Betat[3], Peter F. Stadler[1,4−8], Mario Mörl[3], Jörg Fallmann[1*],

1 Bioinformatics Group, Department of Computer Science, and Interdisciplinary Center for Bioinformatics, University of Leipzig, Härtelstraße 16-18, D-04107 Leipzig, Germany; {anneh,studla,fall}@bioinf.uni-leipzig.de
2 Helmholtz Institute for Metabolic, Obesity and Vascular Research (HI-MAG) of the Helmholtz Zentrum München at the University of Leipzig and University Hospital Leipzig, Philipp-Rosenthal-Str. 27, D-04103 Leipzig, Germany; anne.hoffmann@helmholtz-muenchen.de
3 Institute for Biochemistry, Leipzig University, Brüderstraße 34, D-04103 Leipzig; {lieselotte.erber,heike.betat,mario.moerl}@uni-leipzig.de
4 German Centre for Integrative Biodiversity Research (iDiv) Halle-Jena-Leipzig, Competence Center for Scalable Data Services and Solutions, and Leipzig Research Center for Civilization Diseases, University Leipzig, Germany
5 Max Planck Institute for Mathematics in the Sciences, Inselstraße 22, D-04103 Leipzig, Germany
6 Institute for Theoretical Chemistry, University of Vienna, Währingerstraße 17, A-1090 Wien, Austria;
7 Facultad de Ciencias, Universidad Nacional de Colombia, Sede Bogotá, Colombia;
8 Santa Fe Institute, 1399 Hyde Park Rd., Santa Fe, NM 87501
* Correspondence: fall@bioinf.uni-leipzig.de

Version May 17, 2021 submitted to Non-coding RNA

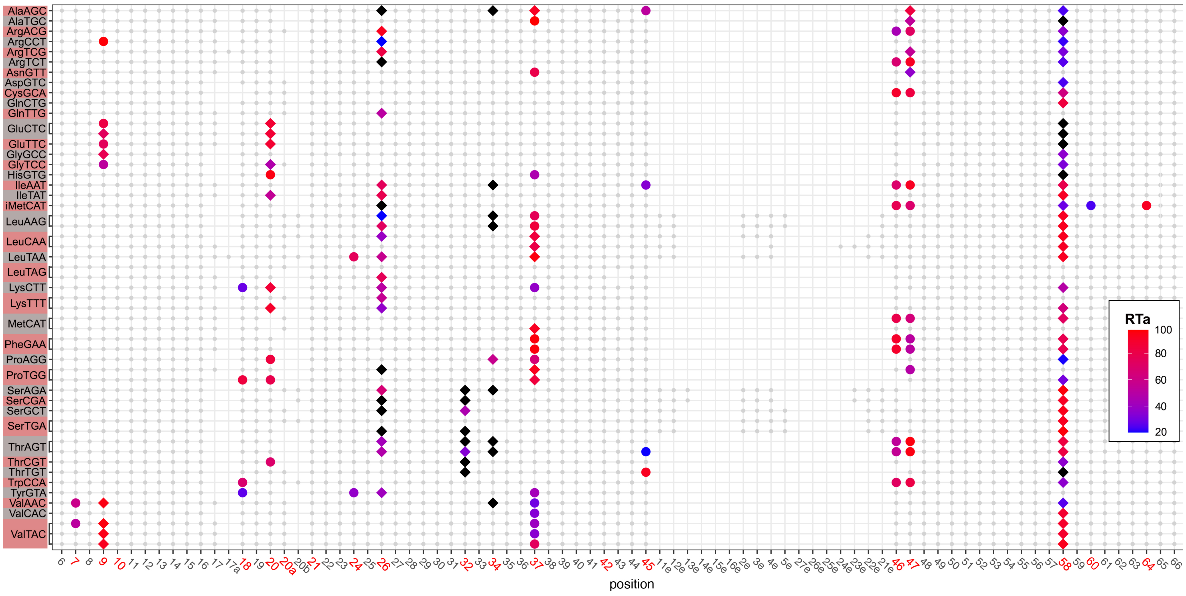

Figure S1. Overview of modification patters in *Dictyostelium discoideum* tRNAs at 6h of the life cycle. tRNAs are ordered by isoacceptor family and anticodon with one line for each distinct tRNA sequence and aligned according to Sprinzl's nomenclature for nucleotide positions [1]. Gray background dots indicate nucleotides present in a sequence. Colored marks indicate the reverse transcriptase arrest (RTa) intensities. Colored ♦ symbolizes tRNA positions that show both RTa and misincoorperation sites, while the symbol in black refers to tRNA positions that show only misincoorperations. • show positions that exhibit only RTa sites in their mapping profile. Only tRNAs with detectable modifications are included. The full set and other time points are given in Tab. S2.

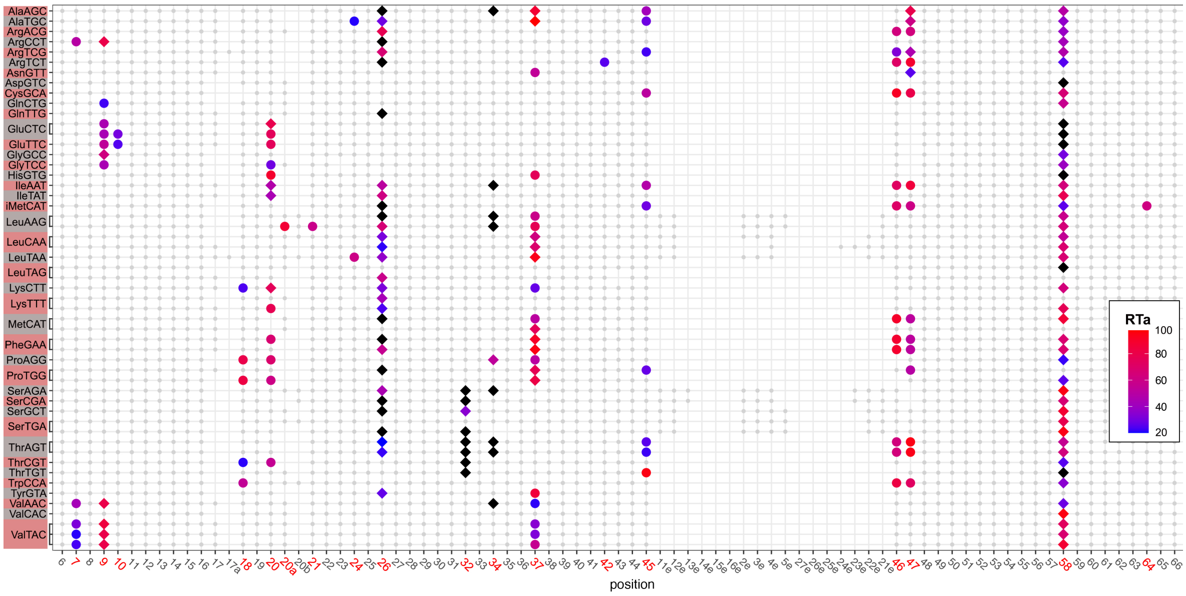

Figure S2. Overview of modification patterns in *Dictyostelium discoideum* tRNAs are shown for the time 16h of the life cycle. The description is according to S1.

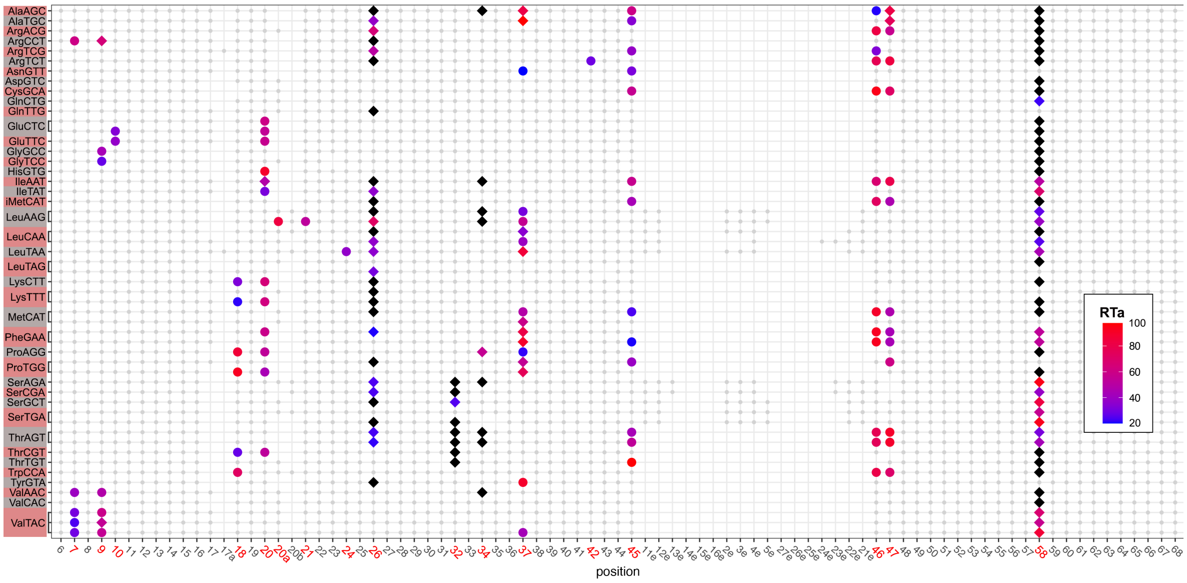

Figure S3. Overview of modification patters in *D. discoideum* tRNAs are shown for the time 20h of the life cycle. The description is according to S1.

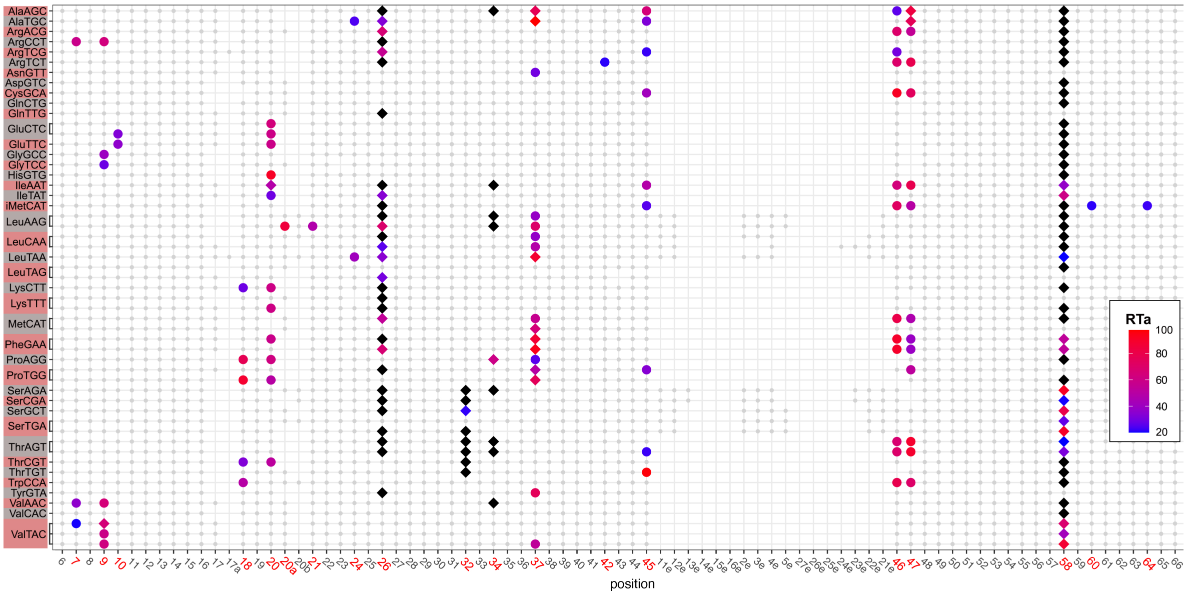

Figure S4. Overview of modification patters in *Dictyostelium discoideum* tRNAs are shown for the time 24h of the life cycle. The description is according to S1.

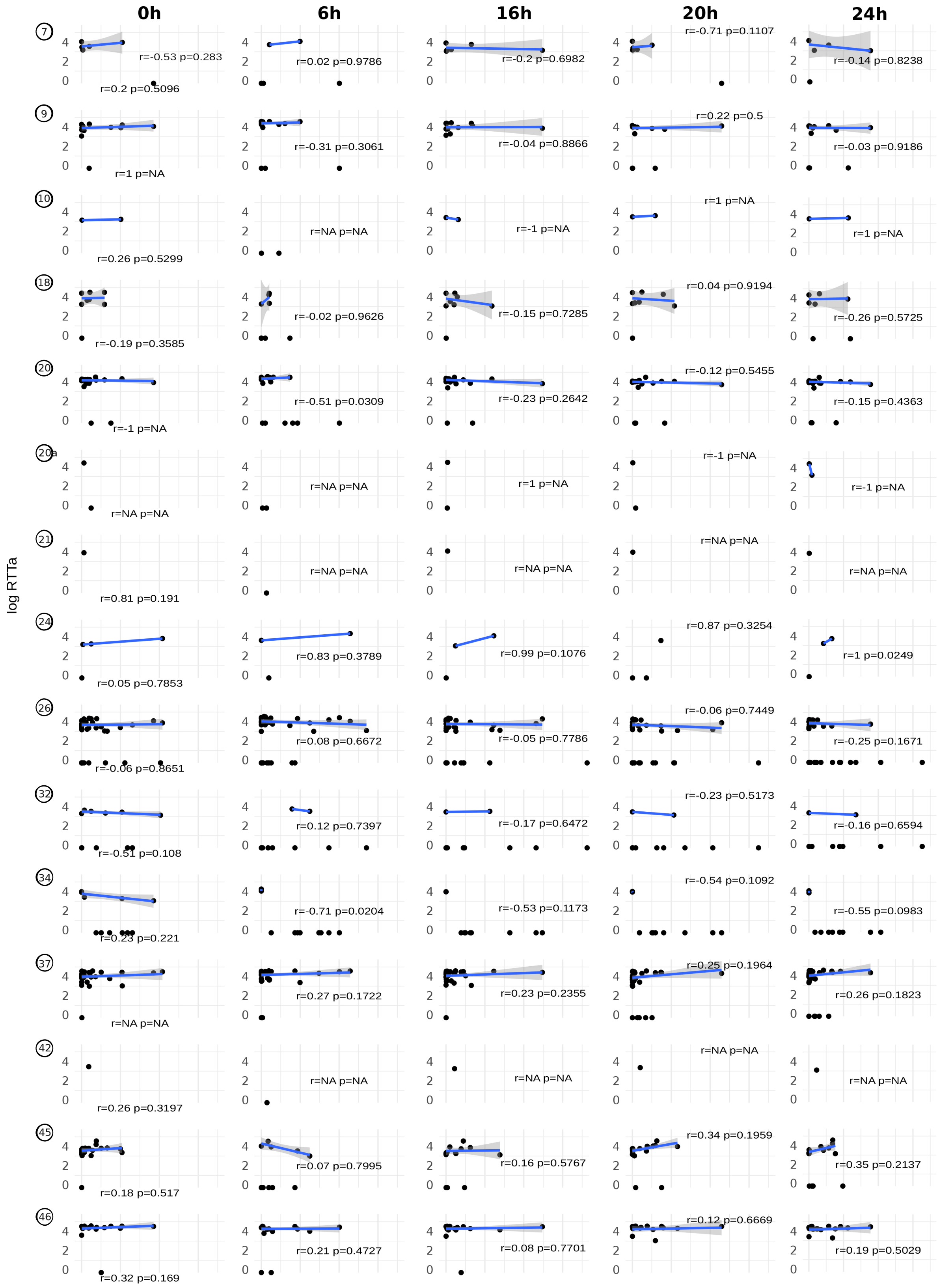

0h
6h
16h
20h
24h
log RTTa
7
9
10
18
20
20a
21
24
26
32
34
37
42
45
46
r=-0.53 p=0.283
r=0.2 p=0.5096
r=0.02 p=0.9786
r=-0.2 p=0.6982
r=-0.71 p=0.1107
r=-0.14 p=0.8238
r=1 p=NA
r=-0.31 p=0.3061
r=-0.04 p=0.8866
r=0.22 p=0.5
r=-0.03 p=0.9186
r=0.26 p=0.5299
r=NA p=NA
r=-1 p=NA
r=1 p=NA
r=1 p=NA
r=-0.19 p=0.3585
r=-0.02 p=0.9626
r=-0.15 p=0.7285
r=0.04 p=0.9194
r=-0.26 p=0.5725
r=-1 p=NA
r=-0.51 p=0.0309
r=-0.23 p=0.2642
r=-0.12 p=0.5455
r=-0.15 p=0.4363
r=NA p=NA
r=NA p=NA
r=1 p=NA
r=-1 p=NA
r=-1 p=NA
r=0.81 p=0.191
r=NA p=NA
r=NA p=NA
r=NA p=NA
r=NA p=NA
r=0.05 p=0.7853
r=0.83 p=0.3789
r=0.99 p=0.1076
r=0.87 p=0.3254
r=1 p=0.0249
r=-0.06 p=0.8651
r=0.08 p=0.6672
r=-0.05 p=0.7786
r=-0.06 p=0.7449
r=-0.25 p=0.1671
r=-0.51 p=0.108
r=0.12 p=0.7397
r=-0.17 p=0.6472
r=-0.23 p=0.5173
r=-0.16 p=0.6594
r=0.23 p=0.221
r=-0.71 p=0.0204
r=-0.53 p=0.1173
r=-0.54 p=0.1092
r=-0.55 p=0.0983
r=NA p=NA
r=0.27 p=0.1722
r=0.23 p=0.2355
r=0.25 p=0.1964
r=0.26 p=0.1823
r=0.26 p=0.3197
r=NA p=NA
r=NA p=NA
r=NA p=NA
r=NA p=NA
r=0.18 p=0.517
r=0.07 p=0.7995
r=0.16 p=0.5767
r=0.34 p=0.1959
r=0.35 p=0.2137
r=0.32 p=0.169
r=0.21 p=0.4727
r=0.08 p=0.7701
r=0.12 p=0.6669
r=0.19 p=0.5029

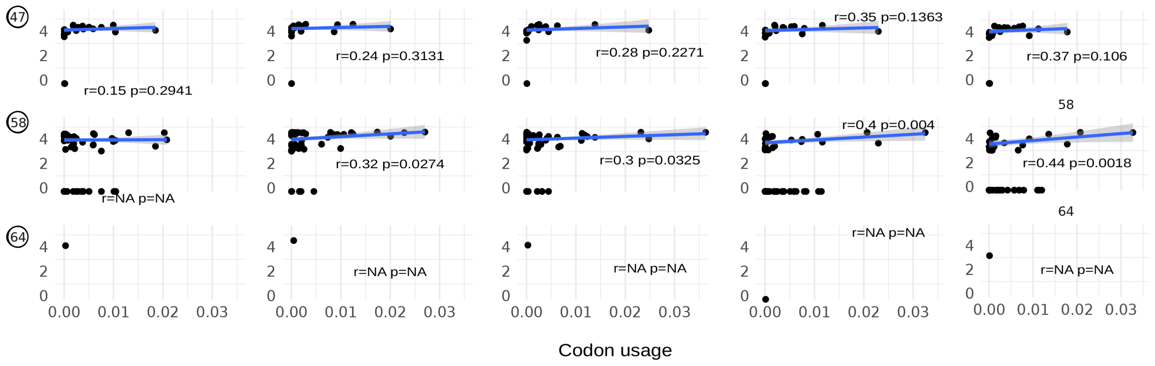

Figure S5. Correlation plots between codon usage and RTa pileup for all investigated developmental stages (0h, 6h, 16h, 20h, and 24h) of *Dictyostelium discoideum* and all positions (circles on the left side) that show modification signature in more than one codon. The number of RTa sites is not significantly correlated with the expression of tRNAs, thus, RTa levels are not confouded by tRNA expression.

Table S1. Collection of reverse transcriptase signatures of tRNA modifications. Modifications that can be classified in *Dictyostelium discoideum* due to their reverse transcriptase signature by analyzing RNA-seq data are listed. Modifications that do not affect our analysis or only become visible by chemical treatment in RNA-seq data are not mentioned. Abbreviations: Misincorporations – MI; Reverse transcriptase arrest – RTa.

Symbol	Name	MI	RTa
acp^3U	3-(3-amino-3-carboxypropyl)uridine	+	+
Cm	2′-*O*-methylcytidine	+	+
D	dihydrouridine	+	+
Gm	2′-*O*-methylguanosine	-	+
I	inosine	+	-
i^6A	N^6-isopentenyladenosine	+	+
m^1A	1-methyladenosine	+	+
m^1G	1-methylguanosine	+	+
m^1I	1-methylinosine	+	-
$m^2{}_2G$	N^2, N^2-dimethylguanosine	+	+
m^2G	N^2-methylguanosine	+	+
m^3C	3-methylcytidine	+	+
m^6t^6A	N^6-methyl-N^6-threonylcarbamoyladenosine	-	+
ms^2i^6A	2-methylthio-N^6-isopentenyladenosine	+	+
o^2yW	peroxywybutosine	+	-
t^6A	N^6-threonylcarbamoyladenosine	+	+

Table S2. Differences regarding the number of modified tRNAs during the life cycle of *Dictyostelium discoideum*. Differences in the amount of modified tRNAs (regarding the individual tRNAs) are given for each position and development stage (0h, 6h, 16h, 20h and 24h after starvation). A development-dependent expression of tRNA modifications is evident, since their number varies greatly at some positions. In particular, the modified tRNAs around 6h after starvation differ most from the other stages. Only modifications at positions C24, A34, T47, and A58 show the same number of modified tRNAs in each of the developmental stages.

Position	Reference nt	0h tRNAs	6h tRNAs	16h tRNAs	20h tRNAs	24h tRNAs
7	A	27	25	45	27	37
7	G	1	-	1	1	1
9	G	92	73	104	81	81
10	G	21	-	21	21	21
18	G	67	32	52	67	34
20	C	35	2	35	35	35
20	G	14	-	14	14	14
20	T	100	88	100	95	95
20a	T	1	-	1	1	7
21	A	1	-	1	1	1
24	C	17	17	17	17	17
24	G	7	1	8	-	8
26	G	241	210	241	240	241
32	C	82	61	61	61	61
34	A	91	91	91	91	91
34	G	33	-	-	-	-
37	A	82	82	89	53	52
37	G	82	83	82	73	74
42	G	11	-	11	11	11
45	G	102	51	81	83	76
46	G	117	116	117	132	132
47	T	142	142	142	140	140
58	A	401	400	401	401	401
60	A	-	8	-	-	8
64	A	8	8	8	-	8

References

1. Sprinzl, M.; Horn, C.; Brown, M.; Ioudovitch, A.; Steinberg, S. Compilation of tRNA sequences and sequences of tRNA genes. *Nucleic Acids Res.* **1998**, *26*, 148–153. doi:10.1093/nar/26.1.148.

© 2021 by the authors. Submitted to *Non-coding RNA* for possible open access publication under the terms and conditions of the Creative Commons Attribution (CC BY) license (http://creativecommons.org/licenses/by/4.0/).
